# Viral-Infected Change of the Digestive Tract Microbiota Associated With Mucosal Immunity in Teleost Fish

**DOI:** 10.3389/fimmu.2019.02878

**Published:** 2019-12-18

**Authors:** Shuai Dong, Li-guo Ding, Jia-feng Cao, Xia Liu, Hao-yue Xu, Kai-feng Meng, Yong-yao Yu, Qingchao Wang, Zhen Xu

**Affiliations:** ^1^Department of Aquatic Animal Medicine, College of Fisheries, Huazhong Agricultural University, Wuhan, China; ^2^Laboratory for Marine Biology and Biotechnology, Qingdao National Laboratory for Marine Science and Technology, Qingdao, China

**Keywords:** digestive tract, microbiota, mucosal immunity, infectious hematopoietic necrosis virus, rainbow trout (*Oncorhynchus mykiss*)

## Abstract

The digestive tract is a unique series of organs that is inhabited by a range of commensal microbes while also exposed to an overwhelming load of dietary antigens. It is widely known that mammals have evolved complex and efficient immune strategies to protect the mucosa of the digestive tract. However, in the early vertebrates, the roles of mucosal immune defense and microbial communities in the different segments of the digestive tract are not well-understood. Here, we constructed a bath infection model with infectious hematopoietic necrosis virus (IHNV) in rainbow trout (*Oncorhynchus mykiss*). Importantly, following viral infection, we found that the IHNV distribution and the reactions of immune-related genes had similar trends that decreased across the digestive tract. Hematoxylin and eosin (H & E) and alcian blue (A & B) staining of the trout digestive tract showed that the pathological changes only occurred in the buccal and pharyngeal mucosal tissues. Moreover, the increased diversity of the microbial community was only detected in the buccal mucosa through 16S rRNA gene sequencing, suggesting that the magnitude of the immune response and microbial community changes are related to the IHNV load and the original microbial diversity. In addition, the loss of digestive tract dominant species and increased colonization of opportunistic bacteria were discovered in the buccal mucosal surface indicating that a secondary bacterial infection occurred in this mucosal tissue.

## Introduction

The digestive tract of vertebrates is responsible for ingesting foods, absorbing nutrition and excreting waste, and therefore it is in continual contact with a complex community of microbiota and environmental pathogens. As digestive mucosal surfaces represent the main sites in which both environmental antigens and microbiota interact with the host, mucosa-associated lymphoid tissue (MALT) has evolved with a complex and efficient immune system to recognize the non-pathogenic microbiota and prevent the invasion of pathogens (1). In mammals, nasopharynx-associated lymphoid tissue (NALT) and gut-associated lymphoid tissue (GALT) and their roles in maintaining mucosal homeostasis in the digestive tract have been well-investigated. NALT includes the adenoids, palatine tonsils, and lingual tonsils, as well as a diffuse network of immune cells (D-NALT) (2, 3). GALT, which consists of scattered lymphocytes and organized lymphoid tissues contain Payer's patches (PP), isolated lymphoid follicles and mesenteric lymph nodes (1). Based on these, both NALT and GALT form the first immune defense line in the oropharyngeal cavity and gastrointestinal (GI) tract (3). In contrast to mammals, teleost NALT has only been described as D-NALT in the olfactory organ, while teleost fish lack the choana, and thus the oropharyngeal cavity is a separate compartment from the nasal cavity, but MALT in the teleost pharynx as well as mucosal immune system is poorly investigated. Moreover, the teleost gut lacks the organized GALT and PP but contains a variety of immune cells, including macrophages, granulocytes, lymphocytes, and plasma cells, in a scattered manner along the alimentary canal (4, 5). Critically, innate and adaptive immune responses can be elicited by immune cells on the teleost posterior by pathogens as in mammals even though they possess different structures (5). It is well-known that in the area at the front of the mammal digestive tract, such as the buccal cavity, secretory IgA (sIgA) mainly from the salivary glands plays an important role in the interaction with oral-associated microbiota and protection of the buccal surface against pathogens (6). However, whether the mucosal immune system presents and contains converted role of mucosal immunoglobulin in the oropharyngeal mucosa remains to be determined. Moreover, the type of oropharyngeal microbiota species and the interaction between microbiota and the host immune system in teleosts are still unknown.

On the mucosal surface of the digestive tracts of vertebrates, abundant microbes are allowed to colonize at high densities. Under homeostatic conditions, primordial commensal microbes impart specific functions in host nutrient, xenobiotic, and drug metabolism and mucosal barrier function, which are essential for the host's health. It is noted that the responses of T cells to microbes in the intestine are suppressed to prevent inappropriate inflammation (7). Moreover, the antimicrobial peptides (AMPs) secreted by the host help to maintain distance between the bacteria and the epithelium (7, 8), and the commensals conversely protect the host's health by extracting nutrients, mediating the immune system, and inhibiting pathogen invasions (7, 9, 10). However, when conditions in the host are disrupted by environmental pathogens, the relationship between the microbiota and the host's immune system shifts significantly that renders commensals into opportunists or pathogens. In humans, the lung-tropic viral influenza infection can lead to *Escherichia coli* overgrowth and cause intestinal immune injury (11). In mice, the loss of transcription factor T-bet can influence the bacterial population in the colon and result in colitis (12). Owing to the development of sequencing technology, many microbe studies have made great progress in mammals and birds. However, although a previous study has found that the intestinal microbiota was dramatically altered in gibel carp after CyHV-2 infection (13), the changes between microbial community and digestive tract immune system under viral infections in teleost are still rarely mentioned and require in-depth studies.

In this study, we examined the immune response and microbial community changes in the different segments of the digestive tract of rainbow trout (*Oncorhynchus mykiss*), including the buccal mucosa, pharynx, stomach, foregut, midgut, and hindgut, after an infectious hematopoietic necrosis virus (IHNV) immersion infection. Our results showed that the IHNV successfully invaded the trout and that the virus loads decreased across the digestive tract. IHNV can elicit a strong local immune response in the digestive tract and changed the original microbial community in the buccal cavity, suggesting that the degree of the immune response and microbial community changes are associated with the IHNV loads and the original microbial diversity. Moreover, IHNV can cause microbial dysbiosis at the buccal mucosal surface, leading to the invasion of opportunistic pathogens, suggesting that secondary bacterial infection might occur at the mucosa of the mouth after viral infection.

## Materials and Methods

### Fish Maintenance

Rainbow trout (10–15 g) were obtained from a fish farm in Shiyan (Hubei province, China) and maintained in aerated aquarium tanks using a water recirculation system including thermostatic temperature control and extensive biofiltration. Fish were acclimatized for at least 2 weeks at 15°C and fed with commercial trout pellets at a rate of 0.5–1% biomass twice a day (9:00 a.m. and 5:00 p.m.), and feeding was terminated 48 h prior to sacrifice both of the control and infected groups. Animal procedures were approved by the Animal Experiment Committee of Huazhong Agricultural University and carried out according to the relative guidelines.

### Virus and Infection

The Cyprinus carpio epithelioma papillosum cyprini (EPC) cell line was maintained in minimum Eagle's medium (MEM) (Gibco) supplemented with 1% Penicillin-Streptomycin Solution and 10% fetal bovine serum (FBS) at 16°C. The IHNV was gifted from Dr. Hong Liu (Shenzhen Entry-Exit Inspection and Quarantine Bureau). The virus was passed *in vitro* on the EPC cell line, titered, adjust to 1 × 10^9^ pfu ml^−1^ in MEM and held in −80°C until use. The methods used for IHNV infection were described previously by Hamdi Ogut et al. (14) with slight modification. Briefly, fish were bathed with a dose of 6 ml IHNV (1 × 10^9^pfu ml^−1^) in 10 L aeration water for 2 h at 15°C. Then the fish were migrated into the aquarium containing new aquatic water and kept for 7 days. As a control, the same number of fish were maintained in similar tanks and were exposed to the MEM without virus.

### Sample Collection

For sampling, rainbow trout were anesthetized with MS-222 and tissues were collected on the 7th day after infection. For histology and pathology study, six different segments of the digestive tract (mouth, pharynx, stomach, foregut, midgut, and hindgut) were directly removed from both the control and infected fish and immediately fixed in 4% (v/v) neutral buffered paraformaldehyde for at least 24 h. For RNA extraction and quantitative real-time PCR (qRT-PCR), samples including mouth, pharynx, stomach, foregut, midgut, hindgut, spleen, and head kidney were collected in sterile micro-centrifuge tubes. For bacteria 16S rRNA gene sequencing, mucosa-associated bacteria were collected by scraping the mucosal layer with a sterile scalpel. All of these tissues collected for RNA or 16S rRNA gene analyses were immediately frozen with liquid nitrogen and stored at −80°C for further study. A schematic representation of the segments of the digestive tract used in this study is included in Supplementary Figure 1.

### Histology and Light Microscopy Studies

After dissected and fixed in 4% neutral buffered formalin at 4°C, the mouth, pharynx, stomach, foregut, midgut, and hindgut of the trout were then dehydrated in a graded ethanol series, cleaned in xylene, embedded in paraffin, and cut in sections of 5 μm in duplicate with a rotary microtome (MICROM International GmbH, Germany). A copy of the sections were stained with classical hematoxylin and eosin (H & E) and alcian blue (A & B) as described previously (15, 16). Images were acquired in microscope (Olympus, Japan) using the Axiovision software. The thickness of epidermis and the number of mucous cells in the epidermis were measured for evaluating microscopic pathological changes in the buccal mucosa and pharynx. Similarly, the length–width ratios of the stomach, foregut, midgut, and hindgut villi were measured for evaluating microscopic pathological changes. Parameters of each image were measured by three different researchers and a mean was taken to reduce random error.

### RNA Isolation and Quantitative Real-Time PCR Analysis

Total RNA was extracted by homogenization in 1 ml TRIZol (Invitrogen) using steel beads and shaking (60 HZ for 1 min) following the manufacturer's instructions. A spectrophotometry (NanoPhotometer NP 80 Touch) was used to quantitate the extracted RNA and agarose gel electrophoresis was used to determine the integrity of the RNA. Equivalent amounts of the total RNA (1,000 ng) were used for cDNA synthesis with the SuperScript first-strand synthesis system for qPCR (YEASEN) in a 20 μl reaction volume. The synthesized cDNA was diluted 3 times and then was used as a template for qRT-PCR analysis.

The qRT-PCR was performed on a qTOWER^3^G PCR system (Analytik Jena AG, Germany) using the EvaGreen 2 × qPCR Master mix (YEASEN). All samples were performed under the following conditions: 95°C for 5 min, followed by 40 cycles at 95°C for 10 s and at 58°C for 30 s. A dissociation protocol was carried out after thermos cycling to confirm that a band of the correct size was amplified. The relative expression levels of immune-related genes were normalized to the mean Ct value of EF1α, β-actin and 18S rRNA by using the 2^−ΔΔCt^ method. Student's *t*-test was conducted using GraphPad 5.0. The results were obtained from three independent experiments and each was performed in triplicate. The primers used for qRT-PCR are listed in Table 1.

**Table 1:** Gene-specific primers used for quantitative real-time PCR in this study.

**Gene**	**GenBank** **accession no**.	**Tm** **(^**°**^C)**	**Amplicon** **length (bp)**	**Primer Sequence (5**^****′****^**-3**^****′****^**)**	
				**Forward primer**	**Reverse primer**
IFNAR	AGO14285.1	58	179	CAGAGCCTCAGGAAGAACT	CAAGGGGTAGAAGAGCATA
MX1	XM_021567440.1	58	72	GATGCTGCACCTCAAGTCCTACTA	CGGATCACCATGGGAATCTGA
TRIM25	ACN11344.1	58	234	AAAGATTCACCCCAAAACC	AAGGCAGGGGAATCATAGT
CXCL9	NP_001268281.1	58	262	GTGGTTTTGCTGGGAGTTT	TTTTGCTTGTCGTCCTTGT
STAT1	NP_001118179.1	58	256	CTCATCCCCTGGACCAAGTT	TTATTGTAGCCCTCCACCCA
MyD88	CDG03206.1	58	192	CAGGACCCAACACGGAAGAT	CCACCTCAGGAACCTGGACT
LGP2	ALE66118.1	58	224	AGTTTGGCACGCAGGAGTA	CAAGCAGGAAGAAGTCGGT
RIG-I	AGN48009.1	58	101	CAGAGGTACTACAGGAAATGG	TTACTGGTCTTCAAGCAATG
CCL19	AIN40032.1	58	139	GTTTCCCTCGCCACTTCAA	GCCACCCACTTGCTCTTTG
MDA5	NP_001182108.1	58	136	CAGTGGAGATGACGATGGG	ACTTGGCGTTCTTGTGCTT
TNFα	XM_021576327.1	58	204	GTATGCGATGACACCTGAA	GCCCCATTAGAGTGCCTTA
IgM	S63348.1	58	157	AAGAAAGCCTACAAGAGGGAGA	CGTCAACAAGCCAAGCCACTA
IgT	AY870264	58		CAGACAACAGCACCTCACCTA	GAGTCAATAAGAAGACACAACGA
IgD	AY748802.1	58	138	CAGGAGGAAAGTTCGGCATCA	CCTCAAGGAGCTCTGGTTTGGA
pIgR	FJ940682.1	58	145	GTACAGCAGGTGTTCACAGTAAC	CCACAGACAGACCTTGGATAAC
C1S	XM_021624859.1	58	176	TGAACAACCTGAACACCCC	CAGCCTATTAGCCTGTAACTCC
C3	XM_021561577.1	58	213	CCTCACAACAAGAGTGCACATC	CCAAGTGGGCAAACTCATCTCC
CATH1	NM_001124480.1	58	152	CTGGAGGCAAGCAACAAC	CCCCCAAGACGAGAGACA
CATH2	AY542963	58	319	ACATGGAGGCAGAAGTTCAGAAGA	GAGCCAAACCCAGGACGAGA
HP	XM_021595153.1	58	164	CGGAGGAGGTTGGAAGC	GCAGCAGAAGCCACAGC
Toll5S	NM_001124208.1	58	139	CATCGCCCTGCAGATTTTAT	CAGAGAGACGAGGCCTTTGA
IL1β	AJ223954.1	58	592	GGCGCGGGGGTTACCATGGGAACCG	GGCGGTTGGGGGCTGCCTTCTGACA
IL8	NM_001124362.1	58	197	TGTCGTTGTGCTCCTGG	CCTGACCGCTCTTGCTC
EF-1α	NM_001124339.1	58	327	CAACGATATCCGTCGTGGCA	ACAGCGAAACGACCAAGAGG
IHNV	M16023.1	58	693	AGAGATCCCTACACCAGAGAC	GGTGGTGTTGTTTCCGTGCAA
β-actin	XM_021592674.1	58	174	GTCACCAACTGGGACGACAT	GTACATGGCAGGGGTGTTGA
18S rRNA	AF308735	58	89	GATCCATTGGAGGGCAAGTCT	CGAGCTTTTTAACTGCAGCAACTTT

*The primers of IgT were obtained from the previous study of Tacchi et al. (17)*.

### DNA Extraction and PCR Amplification

Microbial DNA was extracted from 56 samples using the E.Z.N.A.® soil DNA Kit (Omega Bio-tek, Norcross, GA, USA) according to manufacturer's protocols. The final DNA concentration and purification were determined by NanoDrop 2000 UV-vis spectrophotometer (Thermo Scientific, Wilmington, USA), and DNA quality was checked by 1% agarose gel electrophoresis. The universal primer set 338F (5′-ACTCCTACGGGAGGCAGCAG-3′) and 806R (5′-GGACTACNNGGGTATCTAAT-3′) incorporated specific barcodes and used for the amplification of the V3-V4 hypervariable region of bacterial 16S rRNA genes by thermocycler PCR system (GeneAmp 9700, ABI, USA). The PCR reactions were conducted using the following programs: 3 min of denaturation at 95°C, 27 cycles of 30 s at 95°C, 30 s for annealing at 55°C, and 45 s for elongation at 72°C, and a final extension at 72°C for 10 min. PCR reactions were performed in triplicate 20 μl mixture containing 4 μl of 5 × FastPfu Buffer, 2 μl of 2.5 mM dNTPs, 0.8 μl of each primer (5 μM), 0.4 μl of FastPfu Polymerase, and 10 ng of template DNA. The resulting PCR products were extracted from a 2% agarose gel and further purified using the AxyPrep DNA Gel Extraction Kit (Axygen Biosciences, Union City, CA, USA) and quantified using QuantiFluor™-ST (Promega, USA) according to the manufacturer's protocol.

### Illumina MiSeq Sequencing and Analyses

Purified amplicons were pooled in equimolar and paired-end sequenced (2 × 300) on an Illumina MiSeq platform (Illumina, San Diego, USA) according to the standard protocols by Majorbio Bio-Pharm Technology Co. Ltd. (Shanghai, China).

Raw fastq files were demultiplexed, quality-filtered by Trimmomatic (18) and merged by Fast Length Adjustment of Short Reads (FLASH) with the following criteria: (i) The reads were truncated at any site receiving an average quality score <20 over a 50 bp sliding window. (ii) Primers were exactly matched allowing 2 nucleotide mismatching, and reads containing ambiguous bases were removed. (iii) Sequences whose overlap was longer than 10 bp were merged according to their overlap sequence. Operational taxonomic units (OTUs) were clustered with 97% similarity cutoff using UPARSE (version 7.1 http://drive5.com/uparse/) and chimeric sequences were identified and removed using UCHIME. The taxonomy of each 16S rRNA gene sequence was analyzed by Ribosomal Database Project (RDP) Classifier algorithm (http://rdp.cme.msu.edu/) against the Silva (SSU123) 16S rRNA database using confidence threshold of 70%.

For Lefse analysis, non-parametric factor Kruskal–Wallis rank sum test is applied for determining the species which showed significant difference in abundance. By linear discrimination analysis (LDA), the effect of the different species was estimated.

### Standard Curve for Infectious Hematopoietic Necrosis Virus

For standard curve, pMD 19-T vector containing the IHNV cDNA insert was prepared from recombinant DH5α *Escherichia coli* cells. Plasmid DNA was isolated from an overnight selective culture using HiPure Plasmid Micro Kit (OMEGA). A dilution series of the Plasmid DNA (1.16 × 10^9^ copies/μl to 1.16 × 10^5^ copies/μl) was then prepared and the mean Ct value of each 10-fold dilution was taken for obtaining the standard curve (Supplementary Figure 2). The Ct values of the samples were extrapolated into the standard curve to calculate the copy number.

### Statistical Analysis

An unpaired Student's *t*-test (Prism version 6.0; GraphPad) was used for gene expression and histology data analysis. For 16S analysis, Mann-Whitney test was used to evaluate the differences between control and infection groups. *P-*values of 0.05 or less were considered statistically significant.

### Availability of Data and Material

The NCBI Sequence Read Archive (SRA) accession number for the amplicon data reported in this manuscript is PRJNA588140.

## Results

### Infectious Hematopoietic Necrosis Virus Infection Induced Immune Gene Expression and Morphological Changes in Trout Digestive Tract

**Figure 1:**
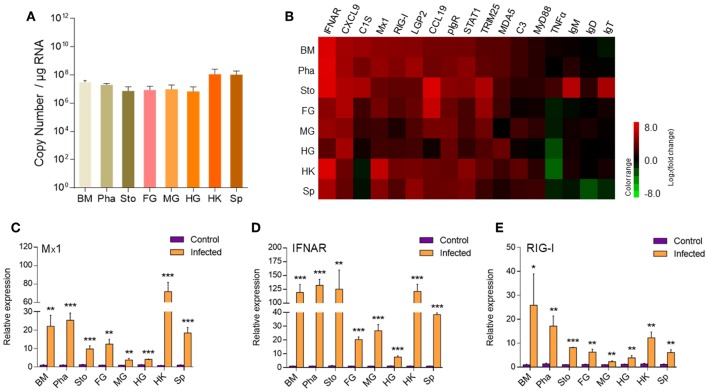
The detection of IHNV and immune-related genes in the buccal mucosa, pharynx, stomach, foregut, midgut, hindgut, head kidney, and spleen of trout at 7 days after infection with IHNV. **(A)** The histogram showed the loads of IHNV in different tissues at 7 dpi (*n* = 6 fish per group). Data are representative of three independent experiments (mean ± SD). **(B)** Heat map illustrates results from quantitative real-time PCR of mRNAs for immune-related genes in virus-challenged fish vs. control fish measured at 7 dpi with IHNV in the buccal mucosa, pharynx, stomach, foregut, midgut, hindgut, head kidney and spleen of rainbow trout (*n* = 6 fish per group). Color value: log_2_ (fold change). The mRNA expression levels of Mx1 **(C)**, IFNAR **(D)**, RIG-I **(E)** were detected at 7 days after infection with IHNV by qRT-PCR (*n* = 6 per group). Control vs. Infected: *p < 0.05, **p < 0.01, ***p < 0.001 (unpaired Student's *t*-test). Data are representative of three independent experiments (mean ± SD). BM, buccal mucosa; Pha, pharynx; Sto, stomach; FG, foregut; MG, midgut; HG, hindgut; HK, head kidney; Sp, spleen.

To assess the immune responses and morphological changes in the digestive tract (mouth, pharynx, stomach, foregut, midgut, and hindgut) of trout, we tested the IHNV bath infection model. The trout were firstly put in a tank containing IHNV for 2 h and then migrated into an aquarium containing new aquatic water where they were kept for 7 days. At 7 days post-infection (dpi), the trout swam lethargically with protruding hemorrhaged eyeballs, typical symptoms of IHNV infection. Then, we sampled the digestive tract (mouth, pharynx, stomach, foregut, midgut, and hindgut) as shown in Supplementary Figure 1. Moreover, by quantitative real-time PCR (qRT-PCR), we detected the copy number of IHNV in the buccal mucosa, pharynx, stomach, foregut, midgut, hindgut, head kidney, and spleen (Figure 1A). As expected, IHNV was detected frequently in the spleen and head kidney. Interestingly, high concentrations of IHNV were observed in the buccal mucosa and pharynx, and a lower IHNV level was detected in the stomach, foregut, midgut, and hindgut. Our results indicated that the loads of IHNV decreased across the digestive tract of trout after infection.

Next using qRT-PCR we measured the expression of 17 immune-related genes, including cytokines (interferon α receptor and tumor necrosis factor α), chemokine genes (CXCL9 and chemokine-like 19), complements (C3 and C1S), tripartite motif protein gene (TRIM25), myxovirus resistance gene (Mx1), activating transcription factor (STAT1), myeloid differentiation factor (MyD88), pattern recognition receptors (RIG-I, LGP2, and MDA5), poly-immunoglobulin receptor (pIgR), and immunoglobulin heavy chain genes (IgT, IgM, and IgD) (Figures 1B–E). Importantly, our studies showed that strong immune responses occurred in the trout buccal mucosa, pharynx, stomach, foregut, midgut, hindgut, head kidney, and spleen after IHNV infection. Critically, upregulated mRNA expression of immune-related genes, such as IFNAR, MX, TRIM25, pIgR, CXCL9, STAT1, LGP2, RIG-I, and MDA5, was detected in every segment of the digestive tract, and these genes showed a stronger reaction in the buccal mucosa and pharynx than in the stomach, foregut, midgut, and hindgut. We selected Mx1, IFNAR, and RIG-I, which are typical anti-virus genes, for the histogram (Figures 1C–E). Mx1 was upregulated ~22-fold in the buccal mucosa and ~25-fold in the pharynx. In contrast, it was upregulated no more than 15-fold in the stomach (~10-fold), foregut (~12-fold), midgut (~four-fold), and hindgut (~four-fold). Similarly, IFNAR was highly induced with ~119-fold in the buccal mucosa, ~132-fold in the pharynx and ~125-fold in the stomach but showed lower fold induction foregut (~20-fold), midgut (~26-fold), and hindgut (~8-fold). Moreover, the expression of RIG-I was also more highly induced in the buccal mucosa (~25-fold) and pharynx (~17-fold) than in the remaining segments of the digestive tract (~8-fold for stomach, ~6-fold for foregut, ~2-fold for midgut, ~4-fold for hindgut). Overall, the decreased intensity of anti-virus genes from the front to the back in the digestive tract showed a similar trend to the distribution of IHNV, which indicated that the intensity of immune reaction was positively related to the abundance of pathogens.

**Figure 2:**
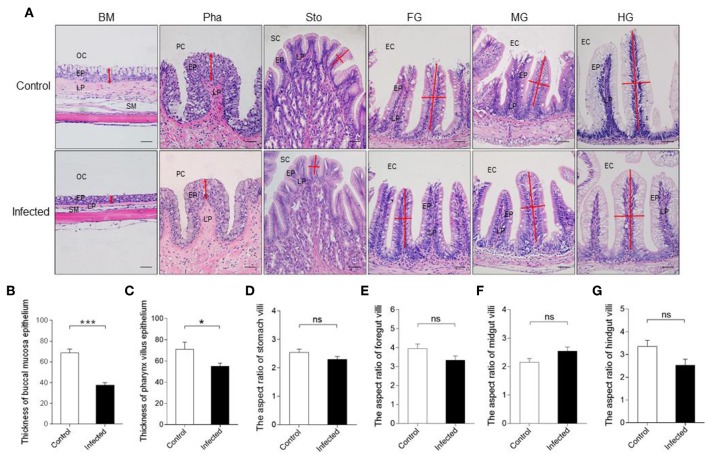
Pathological changes in the digestive tract of rainbow trout following IHNV infection. **(A)** Histological examination (H & E) of the digestive tract (including buccal mucosa, pharynx, stomach, foregut, midgut, and hindgut) from control fish and experimental fish infected with IHNV after 7 days (*n* = 6 fish per group). The thickness of buccal mucosa epidermis **(B)** and pharyngeal villus epidermis **(C)** of control and infected fish (*n* = 6 fish per group). The length-width ratio of stomach villus **(D)**, foregut villus **(E)**, midgut villus **(F)**, and hindgut villus **(G)** of control and infected fish (*n* = 6 fish per group). BM, buccal mucosa; Pha, pharynx; Sto, stomach; FG, foregut; MG, midgut; HG, hindgut; OC, oral cavity; EP, epidermis; LP, lamina propria; SM, submucosa; PC, pharyngeal cavity; SC, stomachic cavity; EC, enteric cavity. The red line with arrowheads represent the thickness of mouth epidermis and pharyngeal villus epidermis. The red line indicates the length or width of stomach villus, foregut villus, midgut villus and hindgut villus. Scale bars, 50 μm. Control vs. Infected: *p < 0.05, ***p < 0.001, ns, not significant, unpaired Student's *t-*test. Data are representative of three different independent experiments (mean ± SEM).

Based on the results of qRT-PCR, we hypothesized that IHNV might cause microscopic pathological changes in the buccal mucosa and pharynx. To verify it, morphological changes were observed in the digestive tract of trout by H & E and A & B staining. Histological examination revealed that the thickness of the epidermis (EP) in the buccal mucosa showed a significant contraction (Figures 2A,B), and the number of mucous cells decreased significantly in the same fish at 7 dpi when compared to the control group (Supplementary Figures 3A,B). Interestingly, similar to the buccal mucosa, we observed a significant decrease of both the thickness of the EP and the number of mucous cells in the pharynx villus (Figures 2A,C and Supplementary Figures 3C,D). However, we did not detect significant differences in the length–width ratios of the stomach, foregut, midgut, and hindgut villi in the infected fish when compared to the control fish (Figures 2A,D–G). Taken together, IHNV infection can cause significant histological changes in the buccal mucosa and pharynx.

### Microbial Dysbiosis Occurred in Buccal Mucosa After Infectious Hematopoietic Necrosis Virus Infection

To evaluate the abundance and diversity changes of the microbiota communities in the digestive tract after IHNV infection, 16S rRNA gene sequencing was conducted using the Illumina MiSeq platform. We obtained 2,551,462 × 2 raw reads from the 56 samples at 7 dpi. Importantly, after removal of the samples with the threshold in M & M mentioned above, a total of 2,371,096 merged sequences were generated, resulting in an average yield of 42,341 (ranging from 26,639 to 61,976) sequences (Supplementary Table 1). Thus, in order to normalize inter-sample variability, all analyses were performed using 26,639 sequences. The resulting sequences with 97% nucleotide sequence identity (97% identity) were binned into operational taxonomic units (OTUs) using UPARSE (19). Based on the RDP classifier, a representative sequence of each OTU was assigned to a taxonomic level in the RDP database using 0.7 as the minimum confidence threshold (version 2.2 http://sourceforge.net/projects/rdp-classifier/).

**Figure 3:**
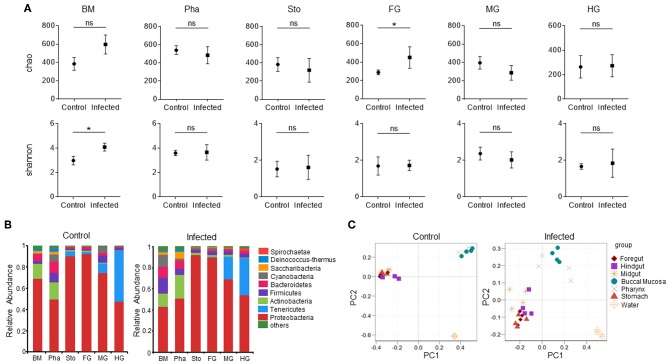
Changes in the abundance and diversity of trout digestive tract microbiota community in response to IHNV infection. **(A)** Richness and diversity of bacterial community in trout buccal mucosa, pharynx, stomach, foregut, midgut and hindgut from control and infected groups (*n* = 4 fish per group). Richness and diversity of the digestive tract bacterial community was measured using Chao mean and Shannon index, respectively. Error bars represent standard error of mean (SEM). Mann-Whitney test was conducted between groups with significance level. Control vs. Infection: *p < 0.05. **(B)** The bacterial community composition in different samples. The relative abundance of bacteria annotated in phylum level. Bar chart of the mean relative abundance in phylum was presented to describe the details. BM, buccal mucosa; Pha, pharynx; Sto, stomach; FG, foregut; MG, midgut; HG, hindgut. **(C)** Principal coordinate analysis (PCoA) with weighted UniFrac distance matrix for all the 56 taxonomic profiles from trout following IHNV infection. Each symbol represents one sample.

Next, we calculated the differences in the microbial abundance and community diversity in the trout digestive tract between the experimental and control groups by using the OTUs noted above for further analysis with UniFrac. Interestingly, the Shannon index for the trout buccal mucosa was higher in the experimental group (Mann–Whitney test, *p* = 0.0286) after IHNV infection than in the control group (Figure 3A, Table 2). In the foregut, we also detected that the Chao1 index significantly increased (*p* = 0.0286), but there were no changes for the Shannon index (*p* = 0.8286). Conversely, we did not find any significant changes in the Chao1 or Shannon indexes in the pharynx, stomach, midgut, or hindgut. Thus, our results indicate that IHNV infection could induce an increase of microbial richness in both the buccal mucosa and foregut. Importantly, IHNV infection could also increase the microbial diversity in the trout buccal mucosa.

**Table 2:** Alpha diversity metrics (mean ± SEM) of the rainbow trout digestive tract microbial community.

	**Buccal mucosa**		**Pharynx**		**Stomach**		**Foregut**		**Midgut**		**Hindgut**	
	**Control**	**Infected**	**Control**	**Infected**	**Control**	**Infected**	**Control**	**Infected**	**Control**	**Infected**	**Control**	**Infected**
Sobs	378.25 ± 40.69	559.75 ± 46.30	522.25 ± 26.32	433.25 ± 55.85	353.50 ± 31.48	298.50 ± 58.75	256.25 ± 8.86	428.25 ± 45.17	366.75 ± 32.47	274.00 ± 36.95	249.75 ± 47.23	258.75 ± 48.71
Shannon	2.99 ± 0.18	4.09 ± 0.16	3.60 ± 0.11	3.65 ± 0.32	1.53 ± 0.21	1.62 ± 0.33	1.69 ± 0.25	1.72 ± 0.14	2.37 ± 0.17	2.02 ± 0.22	1.67 ± 0.07	1.85 ± 0.39
Simpson	0.21 ± 0.04	0.07 ± 0.01	0.09 ± 0.01	0.10 ± 0.02	0.48 ± 0.05	0.47 ± 0.06	0.43 ± 0.07	0.46 ± 0.03	0.28 ± 0.02	0.31 ± 0.02	0.35 ± 0.04	0.31 ± 0.06
Ace	402.70 ± 32.47	594.51 ± 45.67	555.03 ± 23.81	485.87 ± 62.16	412.89 ± 32.64	332.84 ± 64.95	286.87 ± 12.06	471.50 ± 62.03	387.54 ± 31.43	292.90 ± 40.33	270.71 ± 46.13	287.74 ± 47.76
chao	409.34 ± 35.08	612.88 ± 51.60	573.05 ± 24.44	497.85 ± 47.31	411.71 ± 38.00	334.63 ± 65.50	291.73 ± 12.97	479.48 ± 58.28	404.29 ± 34.50	296.19 ± 40.01	275.52 ± 45.72	302.24 ± 45.26
coverage	0.999 ± 0.0003	0.998 ± 0.0002	0.998 ± 0.0001	0.999 ± 0.0009	0.998 ± 0.0002	0.999 ± 0.0004	0.999 ± 0.0002	0.998 ± 0.0007	0.999 ± 0.0003	0.999 ± 0.0003	0.999 ± 0.0001	0.999 ± 0.0001

To further analyze the composition of microbiota in the trout digestive tract, we classified the phylum, class, order, family, and genus of the microbial sequences from the control and infected fish. Our results indicated that for all tissues of the digestive tract from the control group, the predominant bacterial phyla were *Proteobacteria* (mouth: avg. 69.1%, SEM. 6.2%; pharynx: avg. 49.4%, SEM. 3.7%; stomach: avg. 90.2%, SEM. 3.8%; foregut: avg. 92.2%, SEM. 2.9%; midgut: avg. 74.3%, SEM. 4.3%; hindgut: avg. 47.3%, SEM. 9.6%) followed by *Actinobacteria* (mouth and pharynx) or *Tenericutes* (stomach, foregut, midgut and hindgut) (Figure 3B, left). Notably, the abundance of *Proteobacteria* in the trout buccal mucosa dropped (avg. 42.7%, SEM. 3.4%), but there were no significant differences in the other tissues 7 dpi. In addition, *Cyanobacteria* were not the dominant bacteria (avg. 0.4%, SEM. 0.2%) in the buccal mucosa of the control group, but its abundance increased dramatically (avg. 11.3%, SEM. 3.6%) after the IHNV invasion. Principal Coordinate Analysis (PCoA) using the weighted UniFrac distance matrix indicated that the samples of the buccal mucosa and pharynx overlapped, and the samples of the stomach, foregut, midgut, and hindgut overlapped with little separation. Water was separated from these two groups. These results showed the same clustering in both control and infected groups (Figure 3C). In addition, clear separations of the BM microbial communities present between the control and infection group, but not in the microbial communities of the pharynx, stomach, foregut, midgut, and hindgut (Supplementary Figure 4).

### Significantly Changed Microbes of Bacterial Community in Different Groups

**Figure 4:**
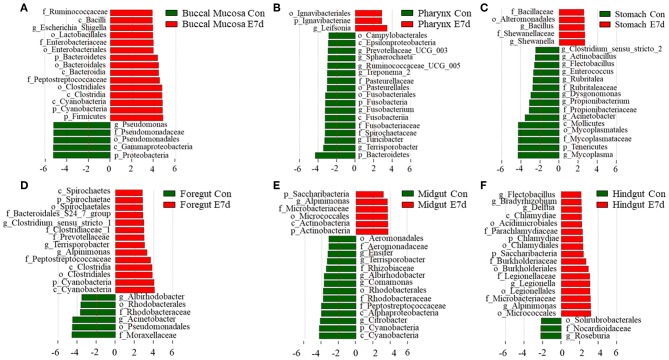
Significantly changed microbes in trout digestive tract after infection with IHNV. Description of top 20 significantly changed microbes that were significant different in the buccal mucosa **(A)**, pharynx **(B)**, stomach **(C)**, foregut **(D)**, midgut **(E)**, and hindgut **(F)** between control and infected groups.

**Figure 5:**
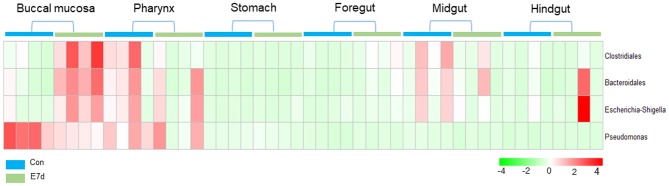
Pheatmap package of R (version 3.4.4) was used to picture the heat map to illustrate the abundance changes of *Pseudomonas, Clostridiales, Bacteroidales*, and *Escherichia-Shigella* in IHNV-challenged and control group. The abundance values were centered and scaled in the row direction.

LDA effect size (LEfSe) analysis was used to further study the significantly changed microbes in different tissues after IHNV infection. Here, we found that the microbe changes in the buccal mucosa were the most significant among the six segments of the digestive tract. We observed that the *Firmicutes* phylum and *Enterobacteriales* family were significantly increased more than 4-fold. In contrast, the *Pseudomonadales* phylum was decreased more than 4-fold (Figure 4A). In the pharynx, stomach, and midgut, only one LDA value of the significantly changed microbes exceeded four. Briefly, the *Bacteroidetes* phylum was obviously decreased in the pharynx. The *Tenericutes* phylum was obviously decreased in the stomach, and the *Cyanobacteria* phylum was significantly decreased in the midgut (Figures 4B–E). In the foregut, the *Pseudomonadales* order was significantly decreased and the *Cyanobacteria* phylum was obviously increased, but the LDA values of the other significantly changed microbes did not exceed four (Figure 4D). In addition, in the hindgut, the LDA values of both the significantly increased microbes and the significantly decreased microbes were <4 (Figure 4F). Based on significant changes of microbes that occurred in the buccal mucosa, we observed that the dominant abundance of *Pseudomonas* belonging to the *Pseudomonadales* phylum was significantly decreased at 7 dpi (Figure 5 and Supplementary Figure 5A). Critically, the pathogenic bacteria of *Clostridiales* and *Bacteroidales*, which belong to *Firmicutes* phylum, were significantly increased, and the pathogenic bacteria *Escherichia-Shigella*, which belong to the Enterobacteriales family, were also significantly increased at 7 dpi (Figure 5 and Supplementary Figures 5B–D). Interestingly, those bacteria did not show significant differences in the pharynx, stomach, foregut, midgut, or hindgut. Although many genera were significantly changed in the digestive tract, the relative abundance was below 1%. Taken together, we found the loss of commensals and increased colonization of opportunistic bacteria in the buccal mucosa.

### Antibacterial Gene Expression in Trout Digestive Tract

**Figure 6:**
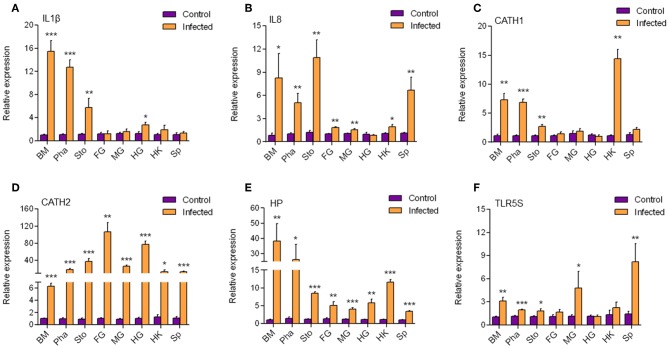
The transcription level of antimicrobial genes in different tissues. The mRNA expression levels of IL1β **(A)**, IL8 **(B)**, CATH1 **(C)**, CATH2 **(D)**, HP **(E)**, and TLR5S **(F)** were detected at 7 day after infection with IHNV by qPCR (*n* = 6 per group). BM, buccal mucosa; Pha, pharynx; Sto, stomach; FG, foregut; MG, midgut; HG, hindgut; HK, head kidney; Sp, spleen. Control vs. Infected: *p < 0.05, **p < 0.01, ***p < 0.001 (unpaired Student *t*-test). Data are representative of three independent experiments (mean ± SD).

According to the results mentioned above, a strong immune reaction and serious pathological changes occurred in the buccal mucosa accompanied by the decrease of dominant species and increase of opportunistic pathogens. Therefore, we hypothesized that a secondary bacterial infection might happen in the buccal mucosa and further detected the expression of antibacterial genes (Figure 6) by qRT-PCR. We found that all six antimicrobial genes including IL1β, IL8, CATH1, CATH2, Hepcidin, and TLR5S were significantly upregulated to various extents in the buccal mucosa, pharynx, and stomach. However, only three, four, and three genes were significantly upregulated in the foregut, midgut, and hindgut, respectively. On the one hand, the decrease of the number of upregulated genes might have been related to the trend of IHNV abundance in the digestive tract. On the other hand, a secondary bacterial infection most likely occurred in the buccal mucosa.

## Discussion

The microbial communities in human mucosa differ significantly according to their location in the body (20). They not only mediate the immune system but also inhibit pathogen invasion (7), which may result in different immune responses when infection occurs. However, whether these interactions occur in the digestive tract of ancient bony vertebrates like teleost fish remains unknown. In order to explore the microbial composition of every segment of the digestive tract and the dynamic changes of the immune responses and microbes after pathogen infection, we used an infection model with IHNV and detected the pathological changes of the rainbow trout digestive tract at 7 dpi. Firstly, we found that the abundance of IHNV decreased across the digestive tract of trout after infection. Interestingly, the expression levels of immune-related genes that we detected in every segment of the digestive tract were in agreement with the distribution of IHNV. Critically, previous studies showed that IFNAR (21), CCL19 (22), Mx1 (23), LGP2 (24), RIG-I (25, 26), and MDA5 (24, 26, 27) were involved in mediating antiviral immunity. We assumed that the more frequent exposure to the virus might have caused the relatively high abundance of IHNV in the buccal mucosa and pharynx and thus the stronger immune responses. In addition, our results showed that the expression levels of IgT, IgM, and IgD did not have any significant changes at 7 dpi. Thus, future studies should be performed at later stages post-pathogen infection to investigate the adaptive immune response in the digestive tract.

Moreover, we detected that the thickness of the mucosa EP at the mouth and the pharynx villus showed significant contraction, and the number of mucous cells in the EP was significantly decreased at the mouth and pharynx villus 7 days post-IHNV challenge. This might have been because the mucous cells at the EP fell off and released mucus including peroxidase (28), lysozyme, immunoglobulins (29), and c-reactive proteins (30, 31) to fight against pathogens. However, in the stomach, foregut, midgut, and hindgut, we discovered that there was no significant difference in the aspect ratio of the gut villus in infected fish when compared to the control trout. Thus, the serious pathological changes were induced by the high relative abundance of IHNV in the buccal mucosa and pharynx. The viral loads in the stomach, foregut, midgut, and hindgut were too low to bring about pathological changes.

In this study, we firstly revealed that the bacterial community in the digestive tract of rainbow trout shifts after IHNV infection. By 16S rRNA sequencing, we found a more diverse bacterial community living in the buccal mucosa and pharynx than that in the stomach, foregut, midgut, and hindgut in naïve fish. A previous study offered insights into the different microbial communities present in the skin, olfactory organ, gills, anterior gut, and posterior gut of rainbow trout (32). It found that the diversity of the bacterial communities in the skin, olfactory organ, and gills is higher than that in the gut. It is common for the skin, olfactory organ, gills, buccal mucosa, and pharynx to be exposed to the environment. Therefore, the teleost mucosa, which directly contacts the external environment, is more suitable for microbial colonization. In the control group, *Proteobacteria* and *Actinobacteria* were dominant in the buccal mucosa and pharynx, while the most abundant phyla of the stomach, foregut, midgut, and hindgut were *Proteobacteria* and *Tenericutes*. This suggests that the core microbiota communities of the buccal mucosa and pharynx differ from those of the stomach, foregut, midgut, and hindgut.

When compared to the infected fish, the bacterial diversity of the mouth significantly increased, whereas the other segments did not have any significant microbiome changes, possibly because the higher viral loads resulted in the increased diversity. Similarly, a recent study revealed that a group infected with a high-dose of SAV3 had a more diverse skin microbiome; however, a low-dose infected group showed an overall loss of bacterial diversity. It indicated that the high-dose infection of SAV3 led to undesired skin colonization of taxa present in the water, including potential pathogens (33). Here, our results further confirmed this suggestion. Because of the decrease of viral load from the buccal mucosa to the hindgut in our results, the degree of the immune response decreased from the mouth to the hindgut. Moreover, the bacterial diversity of the buccal mucosa significantly increased, whereas the other segments did not have any significant microbiome changes. As for the pharynx, the high diversity and rich microbial community among each segment of the digestive tract might have prevented the adhesion of taxa present in the water even if the loads of IHNV at the pharynx were similar to those at the buccal mucosa.

Here, we identified the top 20 bacteria that underwent significant changes in every segment of the digestive tract by LEfSe analysis. Moreover, we showed the significantly decreased abundance of *Pseudomonas* in the buccal mucosa after IHNV infection. In our previous study of skin microbes, we also found the absence of *Pseudomonas* in Ich-infected groups (34). *Pseudomonas* was found to strongly inhibit *Flavobacterium columnare* bacteria in an *in vivo* experiment by preventing the growth of 1,000 times more *F. columnare* cells (35). Thus, *Pseudomonas* plays an important role in the management of opportunistic bacteria, and the loss of *Pseudomonas* during infection might aggravate the condition. Further studies should investigate how the antagonism of *Pseudomonas* against *F. columnare* occurs and whether *Pseudomonas* could play the same protective role in viral infection. In contrast to the decreased abundance of *Pseudomonas*, the increased abundance of *Clostridiales, Bacteroidales*, and *Escherichia-Shigella* was observed in the buccal mucosa. Interestingly, these bacteria are all related to human intestinal diseases. It has been found that *Escherichia-Shigella* can be increased when ulcerative colitis (UC) occurs in the human intestine and then decreased after the patients are treated with 5-aminosalicylic acid (36). As for *Bacteroidetes*, it is the dominant phylum in the human gut (37) but it is also a conditional pathogen. It has been reported that NLRP3, NLRP6, and ASC are important regulators of microbial ecology in mice. The deletion of these genes can increase the number of *Bacteroidetes* (38). In addition, a higher abundance of *Bacteroidetes* in the feces of colorectal cancer patients (38%) is detected when compared to controls (12%) (39). *Clostridium difficile*, a member of the *Clostridiales* order, is the pathogen of *C. difficile* colitis (40). This result suggests that pathogen infection could facilitate colonization of opportunistic bacteria and agrees with the observed increased abundance of *Flavobacteriaceae* in Ich-infected trout skin in our previous research (34). Thus, our results suggest that IHNV infection can induce serious pathological changes and disrupt the steady state of the microecology system in the buccal mucosa. Then, the abundance of dominant species is reduced, and they can no longer protect the mucosal tissue. Finally, the opportunistic bacteria multiply, proliferate, and cause secondary bacterial infections in the mouth.

In order to explore the antimicrobial immune responses that occurred in the buccal mucosa, we detected the expression of six antimicrobial genes in all segments of the digestive tract and found that they were all upregulated in the buccal mucosa. In a previous study, trout IL1β significantly enhanced peritoneal leucocyte phagocytosis to kill the Gram-negative salmonid pathogen *Aeromonas salmonicida* (41). The mRNA expression level of IL1β can be upgraded by CATHs (42), which are AMPs that play an important role during infection with the fish pathogen *Yersinia ruckeri* (43). In addition, CATHs were found to stimulate the mRNA expression of IL8, probably because CATHs signal the recruitment of immune cells *via* IL8 to the site of infection to resolve the infection (43). Hepcidin was reported to protect grass carp against *F. columnare* infection *via* regulating the iron distribution and immune gene expression (44). In the hepatoma cell lines of the rainbow trout RTH-149, stimulation of *Vibrio anguillarum* flagellin allowed the upregulation of rtTLR5S (45), indicating that TLR5S plays an important role in antimicrobial reactions. Therefore, secondary bacterial infection is most likely to occur at the mucosa of the buccal mucosa. Interestingly, HP and CATH2 were also found expressed in the other segments. CATH1, IL8, and IL1β were found mainly expressed in the mouth, pharynx, and stomach. It is possible that secondary infections also occurred in other mucosae.

In summary, we found that the relative abundance of IHNV decreased the across the digestive tract of trout after IHNV infection, and the entire digestive tract exhibited a strong antiviral reaction, especially in the buccal mucosa, pharynx, and stomach. Moreover, the epithelial layer of the trout mouth and pharynx mucosa showed contraction, which was accompanied by the falling off of mucous cells. Through 16S rDNA sequencing of the trout digestive tract, we found that the most diverse microbial community existed in the pharynx. Dysbiosis only occurred in the mouth, and the diversity increased significantly, which was manifested by the decrease of *Pseudomonas* and the increase of *Clostridiales, Bacteroidales*, and *Escherichia-Shigella*. It can be concluded that the magnitude of the immune response and microbial community changes are related to the IHNV loads and the original microbial diversity. The detection of antibacterial genes showed that a strong antibacterial reaction occurred in the digestive tract. This indicates that viral infection can trigger immune responses in the mucosae of the rainbow trout digestive tract and break the micro-ecological balance at the mucosae, leading to the invasion of opportunistic pathogens and secondary infections.

## Data Availability Statement

The SRA accession number for the amplicon data is PRJNA588140. Other raw data supporting the conclusions of this manuscript will be made available by the authors, without undue reservation, to any qualified researcher.

## Ethics Statement

The animal study was reviewed and approved by The Animal Experiment Committee of Huazhong Agricultural University.

## Author Contributions

SD performed most of the experiments, analyzed the data, and wrote the manuscript. LD, JC, XL, HX, and KM help with most of the experiments. YY contributed to setting up the IHNV infection model. ZX and QW designed the experiments and revised the manuscript. All authors reviewed the manuscript.

### Conflict of Interest

The authors declare that the research was conducted in the absence of any commercial or financial relationships that could be construed as a potential conflict of interest.

## References

[1] Mowat AM. Anatomical basis of tolerance and immunity to intestinal antigens. Nat Rev Immunol. (2003) 3:331–41. 10.1038/nri1057 12669023

[2] Perry M Whyte A Immunology of the tonsils. J R Soc Med. (1991) 84:447–8. 10.1177/014107689108400734 PMC1293355 1865465

[3] Sepahi A Salinas I. The evolution of nasal immune systems in vertebrates. Mol Immunol. (2016) 69:131–8. 10.1016/j.molimm.2015.09.008 26391349 PMC6056724

[4] Zimmerman LM Vogel LA Bowden RM. Understanding the vertebrate immune system: insights from the reptilian perspective. J Exp Biol. (2010) 213:661–71. 10.1242/jeb.038315 20154181

[5] Rombout JH Abelli L Picchietti S Scapigliati G Kiron V. Teleost intestinal immunology. Fish Shellfish Immunol. (2011) 31:616–26. 10.1016/j.fsi.2010.09.001 20832474

[6] Brandtzaeg P. Secretory immunity with special reference to the oral cavity. J Oral Microbiol. (2013) 5. 10.3402/jom.v5i0.20401 23487566 PMC3595421

[7] Tuddenham S Sears CL. The intestinal microbiome and health. Curr Opin Infect Dis. (2015) 28:464–70. 10.1097/QCO.0000000000000196 26237547 PMC4643846

[8] Johansson MEV Phillipson M Petersson J Velcich A Holm L Hansson GC. The inner of the two Muc2 mucin-dependent mucus layers in colon is devoid of bacteria. Proc Natl Acad Sci USA. (2008) 105:15064–9. 10.1073/pnas.0803124105 18806221 PMC2567493

[9] Rawls JF Samuel BS Gordon JI. Gnotobiotic zebrafish reveal evolutionarily conserved responses to the gut microbiota. Proc Natl Acad Sci USA. (2004) 101:4596–601. 10.1073/pnas.0400706101 15070763 PMC384792

[10] Rawls JF Mahowald MA Goodman AL Trent CM Gordon JI. *In vivo* imaging and genetic analysis link bacterial motility and symbiosis in the zebrafish gut. Proc Natl Acad Sci USA. (2007) 104:7622–7. 10.1073/pnas.0702386104 17456593 PMC1855277

[11] Wang J Li F Wei H Lian ZX Sun R Tian Z. Respiratory influenza virus infection induces intestinal immune injury via microbiota-mediated Th17 cell-dependent inflammation. J Exp Med. (2014) 211:2397–410. 10.1084/jem.20140625 25366965 PMC4235643

[12] Garrett WS Lord GM Punit S Lugo-Villarino G Mazmanian S Ito S. Communicable ulcerative colitis induced by T-bet deficiency in the innate immune system. Cell. (2007) 131:33–45. 10.1016/j.cell.2007.08.017 17923086 PMC2169385

[13] She R Li TT Luo D Li JB Yin LY Li H. Changes in the Intestinal Microbiota of Gibel Carp (*Carassius gibelio*) associated with cyprinid herpesvirus 2 (CyHV-2) Infection. Curr Microbiol. (2017) 74:1130–6. 10.1007/s00284-017-1294-y 28748273

[14] Ogut H Reno PW Early Kinetics of Infectious Hematopoietic Necrosis Virus (IHNV) Infection in Rainbow Trout. J Aqua Anim Health. (2004) 16:152–60. 10.1577/H03-044.1

[15] Yu YY Kong W Yin YX Dong F Huang ZY Yin GM. Mucosal immunoglobulins protect the olfactory organ of teleost fish against parasitic infection. PLoS Pathog. (2018) 14:e1007251. 10.1371/journal.ppat.1007251 30395648 PMC6237424

[16] Vatsos IN Kotzamanis Y Henry M Angelidis P Alexis M. Monitoring stress in fish by applying image analysis to their skin mucous cells. Eur J Histochem. (2010) 54:22. 10.4081/ejh.2010.e22 20558343 PMC3167306

[17] Tacchi L Musharrafieh R Larragoite ET Crossey K Erhardt EB Martin SAM. Nasal immunity is an ancient arm of the mucosal immune system of vertebrates. Nat Commun. (2014) 5:5205. 10.1038/ncomms6205 25335508 PMC4321879

[18] Bolger AM Lohse M Usadel B. Trimmomatic: a flexible trimmer for Illumina sequence data. Bioinformatics. (2014) 30:2114–20. 10.1093/bioinformatics/btu170 24695404 PMC4103590

[19] Edgar RC. UPARSE: highly accurate OTU sequences from microbial amplicon reads. Nat Methods. (2013) 10:996–8. 10.1038/nmeth.2604 23955772

[20] Robinson CM Pfeiffer JK. Viruses and the Microbiota. Annu Rev Virol. (2014) 1:55–69. 10.1146/annurev-virology-031413-085550 25821837 PMC4373533

[21] Abramovich C Shulman LM Ratovitski E Harroch S Tovey M Eid P. Differential tyrosine phosphorylation of the IFNAR chain of the type I interferon receptor and of an associated surface protein in response to IFN-alpha and IFN-beta. Embo J. (1994) 13:5871–7. 10.1002/j.1460-2075.1994.tb06932.x 7813427 PMC395562

[22] Sepahi A Tacchi L Casadei E Takizawa F LaPatra SE Salinas I. CK12a, a CCL19-like chemokine that orchestrates both nasal and systemic antiviral immune responses in rainbow trout. J Immunol. (2017) 199:3900–13. 10.4049/jimmunol.1700757 29061765 PMC5698097

[23] Trobridge GD Leong JA. Characterization of a rainbow trout Mx gene. J Interferon Cytokine Res. (1995) 15:691–702. 10.1089/jir.1995.15.691 8528941

[24] Chang M Collet B Nie P Lester K Campbell S Secombes CJ. Expression and functional characterization of the RIG-I-like receptors MDA5 and LGP2 in Rainbow trout (*Oncorhynchus mykiss*). J Virol. (2011) 85:8403–12. 10.1128/JVI.00445-10 21680521 PMC3147945

[25] Biacchesi S LeBerre M Lamoureux A Louise Y Lauret E Boudinot P. Mitochondrial antiviral signaling protein plays a major role in induction of the fish innate immune response against RNA and DNA viruses. J Virol. (2009) 83:7815–27. 10.1128/JVI.00404-09 19474100 PMC2715792

[26] Takeuchi O Akira S. MDA5/RIG-I and virus recognition. Curr Opin Immunol. (2008) 20:17–22. 10.1016/j.coi.2008.01.002 18272355

[27] Wang L Su J Yang C Wan Q Peng L. Genomic organization, promoter activity of grass carp MDA5 and the association of its polymorphisms with susceptibility/resistance to grass carp reovirus. Mol Immunol. (2012) 50:236–43. 10.1016/j.molimm.2012.01.012 22361281

[28] Brokken LJS Verbost PM Atsma W Bonga SEW Isolation, partial characterization and localization of integumental peroxidase, a stress-related enzyme in the skin of a teleostean fish (*Cyprinus carpio* L.). Fish Physiol Biochem. (1998) 18:331–42. 10.1023/A:1007707520177

[29] Salinas I Zhang YA Sunyer JO. Mucosal immunoglobulins and B cells of teleost fish. Dev Comp Immunol. (2011) 35:1346–65. 10.1016/j.dci.2011.11.009 22133710 PMC3428141

[30] Shephard RJ Rhind S Shek PN. Exercise and the immune system. Natural killer cells, interleukins and related responses. Sports Med. (1994) 18:340–69. 10.2165/00007256-199418050-00006 7871295

[31] Bhawan J Grimes PA. A histological examination for skin atrophy after 6 months of treatment with fluocinolone acetonide 0.01%, hydroquinone 4%, and tretinoin 0.05% cream. Am J Dermatopathol. (2009) 31:794–8. 10.1097/DAD.0b013e3181a9070d 19755910

[32] Lowrey L Woodhams DC Tacchi L Salinas I. Topographical mapping of the rainbow trout (*Oncorhynchus mykiss*) microbiome reveals a diverse bacterial community with antifungal properties in the skin. Appl Environ Microbiol. (2015) 81:6915–25. 10.1128/AEM.01826-15 26209676 PMC4561705

[33] Reid KM Patel S Robinson AJ Bu L Jarungsriapisit J Moore LJ. Salmonid alphavirus infection causes skin dysbiosis in Atlantic salmon (*Salmo salar L.*) post-smolts. PLoS ONE. (2017) 12:e0172856. 10.1371/journal.pone.0172856 28264056 PMC5338768

[34] Zhang X Ding L Yu Y Kong W Yin Y Huang Z. The change of teleost skin commensal microbiota is associated with skin mucosal transcriptomic responses during parasitic infection by *Ichthyophthirius multifillis*. Front Immunol. (2018) 9:2972. 10.3389/fimmu.2018.02972 30619329 PMC6305302

[35] Tiirola M Valtonen ET Rintamäki-Kinnunen P Kulomaa MS. Diagnosis of *flavobacteriosis* by direct amplification of rRNA genes. Dis Aquatic Organ. (2002) 51:93–100. 10.3354/dao051093 12363090

[36] Xu J Chen N Wu Z Song Y Zhang Y Wu N. 5-aminosalicylic acid alters the gut bacterial microbiota in patients with ulcerative colitis. Front Microbiol. (2018) 9:1274. 10.3389/fmicb.2018.01274 29951050 PMC6008376

[37] Clemente JC Ursell LK Parfrey LW Knight R. The impact of the gut microbiota on human health: an integrative view. Cell. (2012) 148:1258–70. 10.1016/j.cell.2012.01.035 22424233 PMC5050011

[38] Tremaroli V Backhed F. Functional interactions between the gut microbiota and host metabolism. Nature. (2012) 489:242–9. 10.1038/nature11552 22972297

[39] Sears CL. Enterotoxigenic *Bacteroides fragilis*: a rogue among symbiotes. Clin Microbiol Rev. (2009) 22:349–69. 10.1128/CMR.00053-08 19366918 PMC2668231

[40] Ciarán P Kelly M Thomas J LaMont M *Clostridium difficile* infection. Ann Rev Med. (1998) 49:375–90. 10.1146/annurev.med.49.1.375 9509270

[41] Secombes CJ Wang T Bird S. The interleukins of fish. Dev Comp Immunol. (2011) 35:1336–45. 10.1016/j.dci.2011.05.001 21605591

[42] Schmitt P Wacyk J Morales-Lange B Rojas V Guzman F Dixon B. Immunomodulatory effect of cathelicidins in response to a beta-glucan in intestinal epithelial cells from rainbow trout. Dev Comp Immunol. (2015) 51:160–9. 10.1016/j.dci.2015.03.007 25818364

[43] Bridle A Nosworthy E Polinski M Nowak B. Evidence of an antimicrobial-immunomodulatory role of Atlantic salmon cathelicidins during infection with *Yersinia ruckeri*. PLoS ONE. (2011) 6:e23417. 10.1371/journal.pone.0023417 21858109 PMC3153500

[44] Wei X Sarath Babu V Lin L Hu Y Zhang Y Liu X. Hepcidin protects grass carp (*Ctenopharyngodon idellus*) against *Flavobacterium columnare* infection via regulating iron distribution and immune gene expression. Fish Shellfish Immunol. (2018) 75:274–83. 10.1016/j.fsi.2018.02.023 29452250

[45] Tsujita T Tsukada H Nakao M Oshiumi H Matsumoto M Seya T. Sensing bacterial flagellin by membrane and soluble orthologs of Toll-like receptor 5 in rainbow trout (*Onchorhynchus mikiss*). J Biol Chem. (2004) 279:48588–97. 10.1074/jbc.M407634200 15339910

