# Supplementary material for: Viral-Infected Change of the Digestive Tract Microbiota Associated With Mucosal Immunity in Teleost Fish
Source: Front Immunol. 2019 Dec 18;10:2878. doi: 10.3389/fimmu.2019.02878 (PMC6930168; doi:10.3389/fimmu.2019.02878)
Supplement: Supplementary file 1 [file Data_Sheet_1.docx]

**Supplementary Material**:

**TABLE S1 |** Reads Information.

|  | Water | | Mouth | | Pharynx | | Stomach | | Foregut | | Midgut | | Hindgut | |
| --- | --- | --- | --- | --- | --- | --- | --- | --- | --- | --- | --- | --- | --- | --- |
|  | Control | Infected | Control | Infected | Control | Infected | Control | Infected | Control | Infected | Control | Infected | Control | Infected |
| Read Number | 55276 | 53728 | 38252 | 31040 | 36050 | 40468 | 42942 | 41421 | 26639 | 42466 | 35553 | 37977 | 42910 | 41603 |
|  | 54702 | 61976 | 37751 | 34816 | 41329 | 41797 | 44600 | 41942 | 44950 | 44551 | 36555 | 35996 | 43081 | 38210 |
|  | 49854 | 53308 | 40433 | 46794 | 41432 | 35720 | 43477 | 45720 | 41556 | 44288 | 42066 | 38081 | 41689 | 40859 |
|  | 46188 | 55576 | 45420 | 35358 | 41148 | 38714 | 41764 | 39005 | 38919 | 42399 | 36830 | 41193 | 46489 | 48238 |


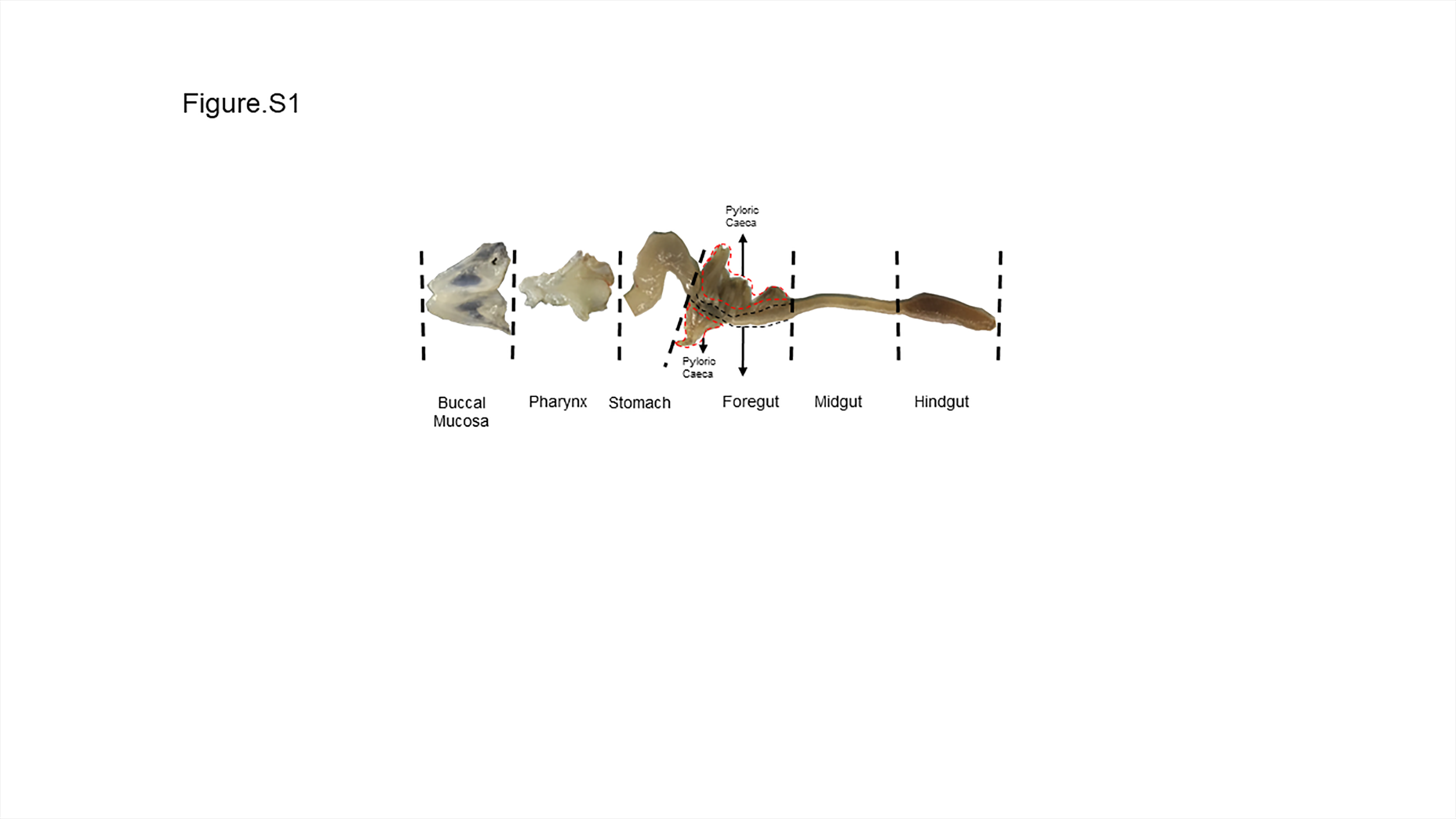


**FIGURE S1 |** Digestive tract segment used in this study. Schematic illustrating of the different segments in the trout digestive tract used in this study.


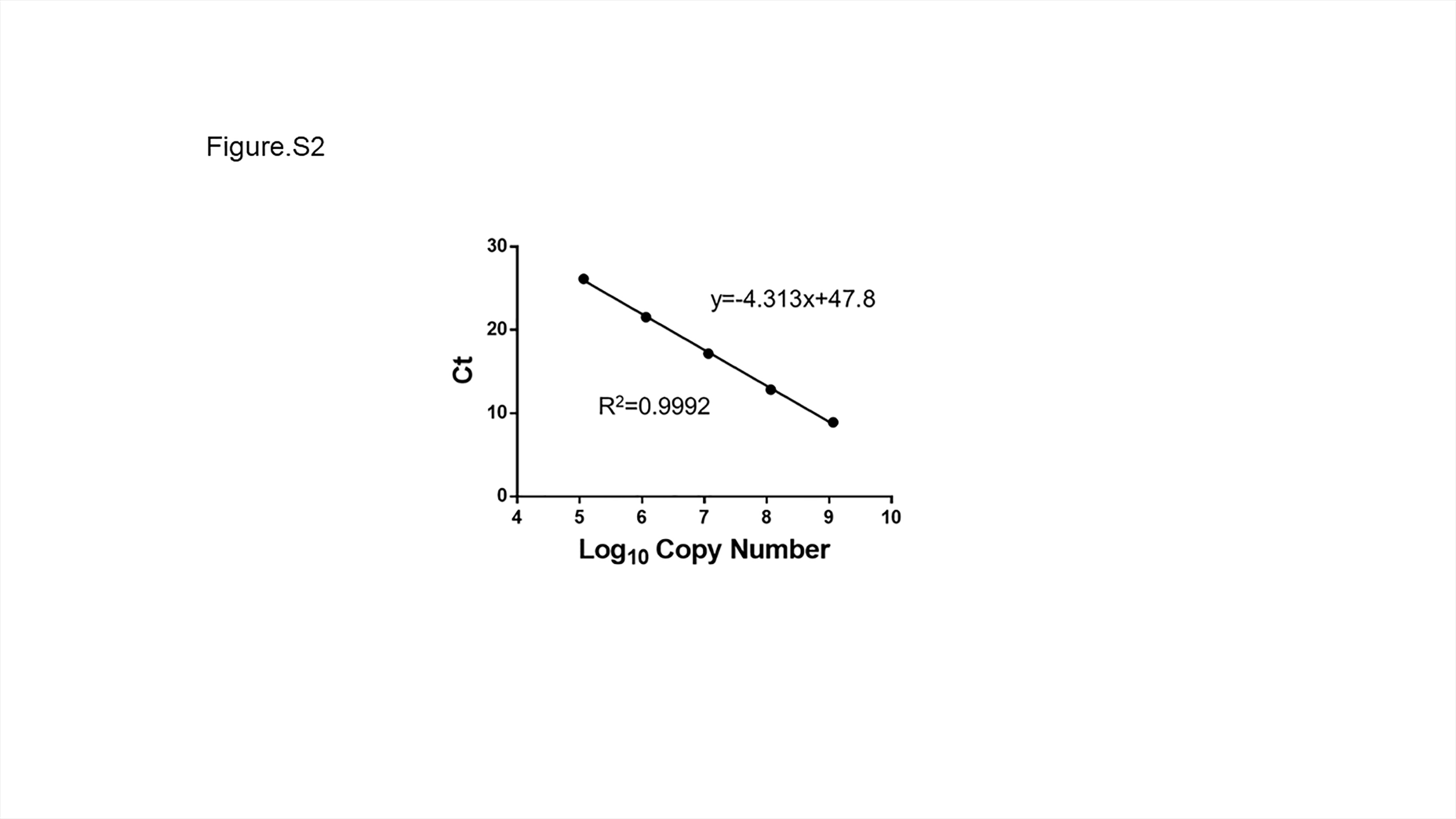


**FIGURE S2 |** Standard curve for IHNV copy number vs cycle threshold (Ct) value. pMD 19-T vector containing the IHNV cDNA insert was serially diluted from 1.16 × 10^9^ copies/μl to 1.16 × 10^5^ copies/μl. Resulting Ct values are plotted against the logarithm of their respective copy numbers.


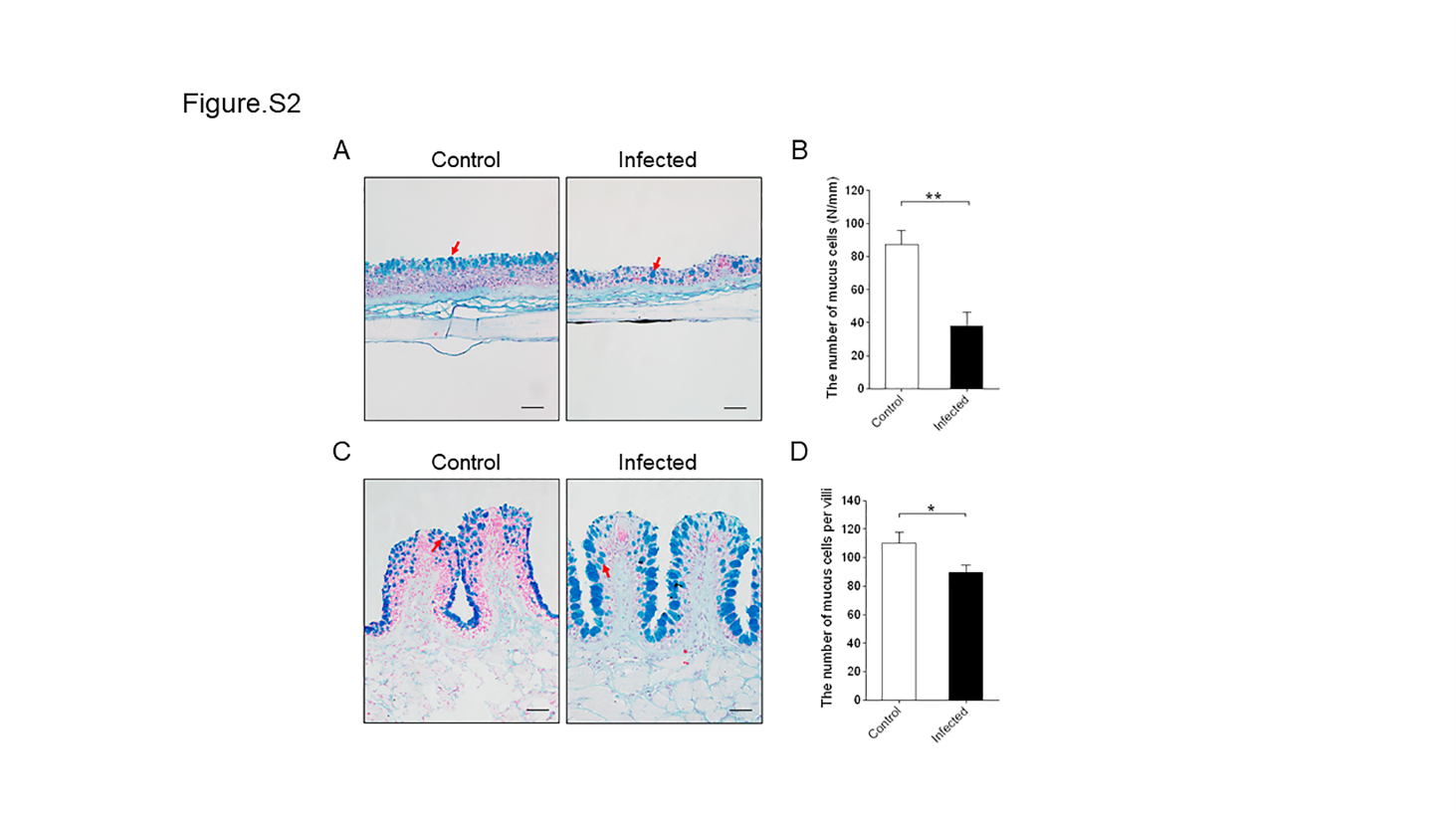


**FIGURE S3 |** Mucous cells in trout buccal mucosa and pharynx. AB stain of buccal mucosa **(A)** and pharynx **(C)** from control and infected fish (*n* = 6 fish per group). The number of mucus cells per millimeter in buccal mucosa epidermis **(B)** and pharyngeal villus epidermis **(D)** of control and infected fish (*n* = 6 fish per group). Red triangles indicate mucus cells. Scale bars, 50 μm. Control vs. Infection: **p* < 0.05, ***p* < 0.01 (unpaired Student *t* test). Data are representative of three different independent experiments (mean ± SEM).


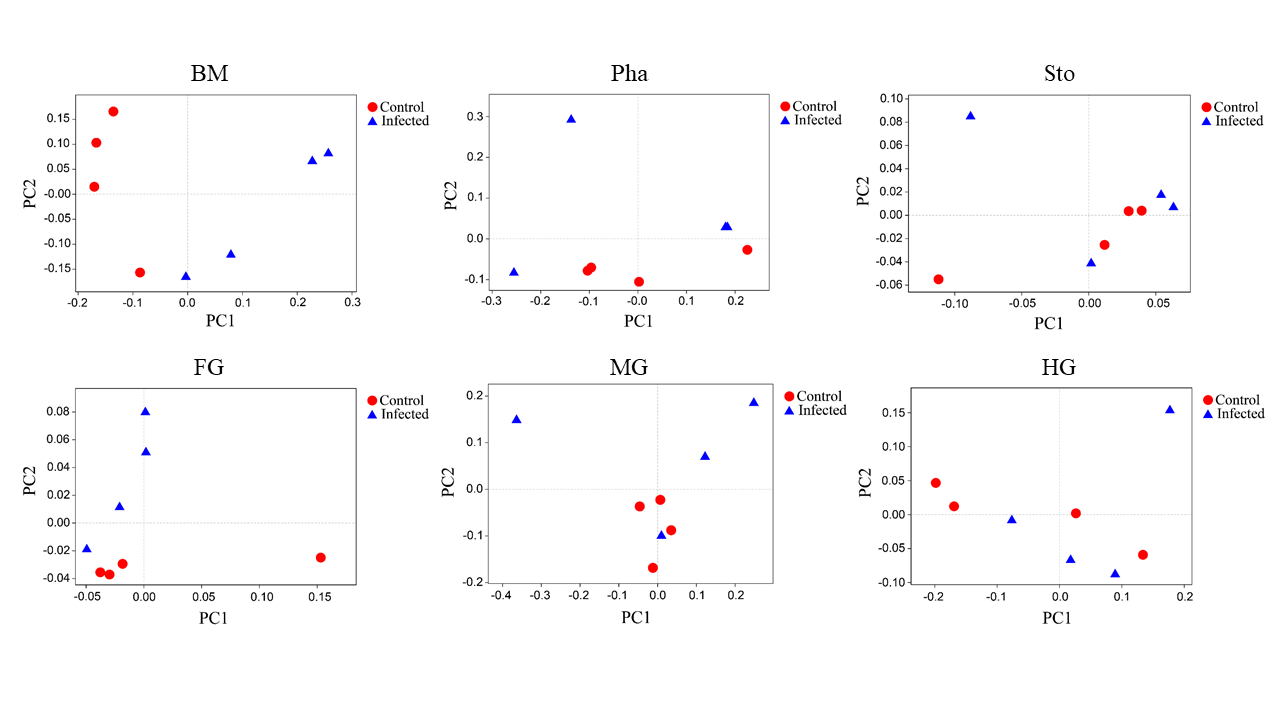


**FIGURE S4 |** Principal coordinate analysis (PCoA) with weighted UniFrac distance matrix for BM (A), Pha (B), Sto (C), FG (D), MG (E) and HG (F). Each symbol represents one sample. BM: buccal mucosa, Pha: pharynx, Sto: stomach, FG: foregut, MG: midgut, HG: hindgut.


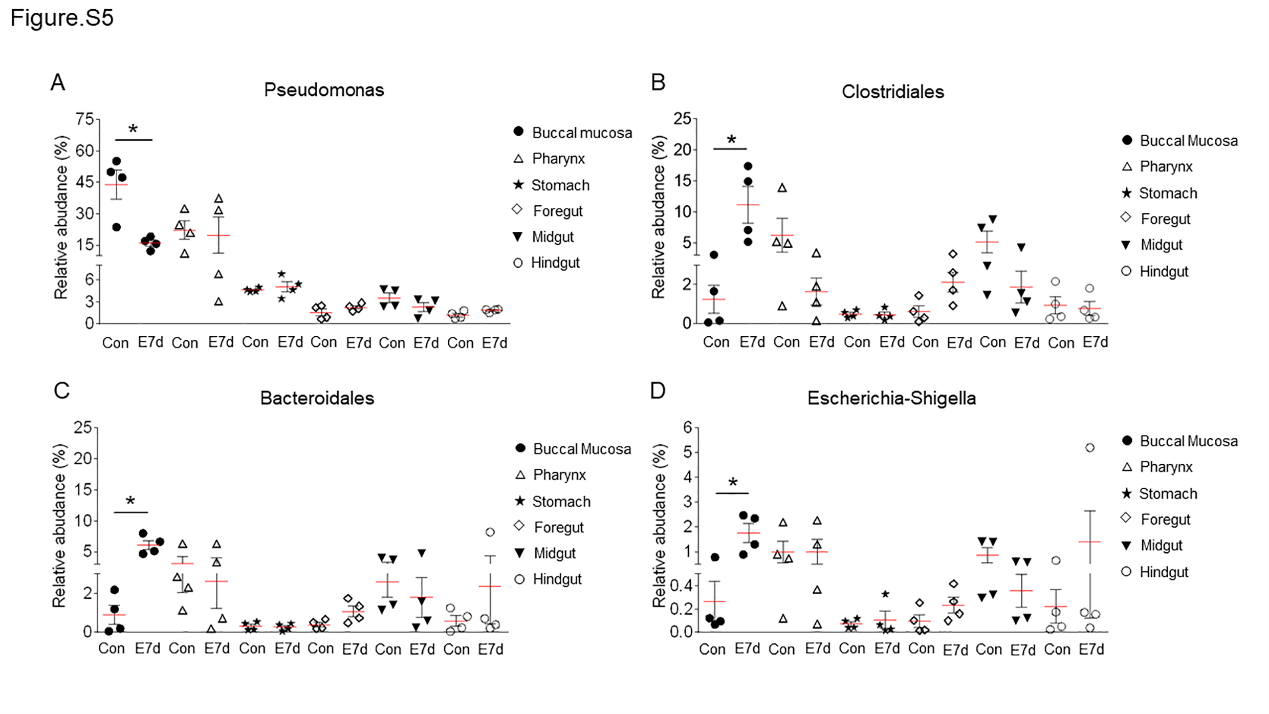


**FIGURE S5 |** IHNV infection results in losses of beneficial bacteria and increased abundance of opportunistic pathogens in trout digestive tract. Percentage of total OUTs represented by *Pseudomonas* **(A)**, *Clostridiales* **(B)**, *Bacteroidales* **(C)** and *Escherichia-Shigella* **(D)** in trout buccal mucosa, pharynx, stomach, foregut, midgut and hindgut between control and infected groups were shown. Con, control. E7d: infected for 7 days. Control vs. Infected: **p* < 0.05, Mann Whitney test.
